# Materials in the Na_2_O–CaO–SiO_2_–P_2_O_5_ System for Medical Applications

**DOI:** 10.3390/ma16175981

**Published:** 2023-08-31

**Authors:** Maksim R. Kaimonov, Tatiana V. Safronova

**Affiliations:** 1 Department of Materials Science, Lomonosov Moscow State University, Leninskie Gory 1, Building 73, 119991 Moscow, Russia; 2 Department of Chemistry, Lomonosov Moscow State University, Leninskie Gory 1, Building 3, 119991 Moscow, Russia

**Keywords:** Bioglass 45S5, bioactive glass–ceramics, calcium phosphates, Na_2_O–CaO–SiO_2_–P_2_O_5_ system, composites

## Abstract

Calcium phosphate materials and materials based on silicon dioxide have been actively studied for more than 50 years due to their high biocompatibility and bioactivity. Hydroxyapatite and tricalcium phosphate are the most known among calcium phosphate materials, and Bioglass 45S5 is the most known material in the Na_2_O–CaO–SiO_2_–P_2_O_5_ system. Each of these materials has its application limits; however, some of them can be eliminated by obtaining composites based on calcium phosphate and bioglass. In this article, we provide an overview of the role of silicon and its compounds, including Bioglass 45S5, consider calcium phosphate materials, talk about the limits of each material, demonstrate the potential of the composites based on them, and show the other ways of obtaining composite ceramics in the Na_2_O–CaO–SiO_2_–P_2_O_5_ system.

## 1. Introduction

The interest in biomaterials (glass, ceramics, glass–ceramics), which include silicon dioxide (SiO_2_), is due to silicon (Si) itself since silicon is one of the main trace elements in the human body; the content in bones reaches 100 ppm and in the extracellular matrix up to 550 ppm [1]. The total silicon content in a human body with a weight of 70 kg ranges from 140 to 700 mg. The silicon compound in the blood is a free orthosilicic acid, which is not bound to proteins. The concentration of this acid can range from 50 to 200 μg/L and depends on the silicon content in the diet [2,3]. In our body, the repositories of silicon are the thyroid gland, adrenal glands, pituitary gland, and lymph nodes. Silicon is usually absorbed as metasilicate, widely distributed in connective tissue [4]. The largest amount of silicon is in hair and nails. It is involved in bone calcification and improving bone density, and a lack of it is one of the causes of osteoporosis. For instance, an experiment on animals [3] showed that silicon increases the rate of mineralization and calcification of bones analogically to vitamin D [5,6]. Vitamin D accelerates the mineralization and formation of bone tissue, so its deficiency limits bone development. However, under conditions of silicon deficiency, a low level of calcification and collagen formation is observed, regardless of the level of vitamin D. Thus, high silicon content in new bone allows for an increasing calcification degree in the early stages of osteogenesis, whilst a silicon deficiency always causes bone distortion [3,7]. In addition, silicon in the form of orthosilicic acid stimulates the synthesis of type I collagen, osteoblasts, and skin fibroblasts; it increases the extent of bone differentiation of Mg-63 cells in vitro [8]. The role of orthosilicic acid is to modulate the activity of the enzyme propyl hydroxylase, which is involved in collagen production [3,9].

Some studies have shown that silicon present in a material can not only stimulate collagen and proteoglycan synthesis but also regulate the expression of genes associated with bones [10]. It has been reported that ionic products released during the degradation of calcium silicate ceramics can increase the effectiveness of insulin-like growth factor 2, which is in a certain way associated with cell proliferation [11]. This enhancement is achieved by inducing transcriptional growth factors and carrier proteins, both regulating the separation of binding proteins. In addition, the properties of silica gel formed in the presence of microorganisms and enzymes are also quite interesting. If the formation of silica gel occurs assisted by Bacillus mycoides, it accelerates the growth of these microorganisms. If silica gel forms assisted by enzymes, it exhibits biocatalyst properties [3,12].

Thus, silicon ions affect human bone metabolism and angiogenesis [3,7,8,9,10,11,12]; they are essential for metabolic processes, and in the early stages of osteogenesis, high silicon content in new bone allows an increase in the degree of calcification; helps improve bone mineral density; and in physiological solutions causes precipitation of hydroxyapatite and hydroxyl carbonate apatite—the basis of bone tissue includes calcium and phosphorous ions.

Calcium ions are the main constituent of bones and teeth; they are involved in the processes of nervous tissue excitability, muscle contractility, and blood clotting; constitute a part of nucleus and cell membranes and cellular and tissue fluids; possess anti-allergic and anti-inflammatory effects; activate some enzymes and hormones [13]; favor osteoblast proliferation, differentiation, and extracellular matrix (ECM) mineralization; activate Ca-sensing receptors in osteoblast cells; and increase expression of growth factors, including insulin-like growth factor (IGF-I or IGF-II) [14].

Phosphorous ions are the main constituent of bones and teeth; they participate in the synthesis of nucleic acids, proteins, and adenosine triphosphate (ATP); produce a source of energy, forming energy-rich phosphate bonds in cellular processes (adenosine diphosphate (ADP) + PO_4_^3−^↔ATP); constitute a part of phospholipids of cell membranes; and stimulate expression of matrix Gla protein (MGP), a key regulator in bone formation [13].

Hydroxyapatite is a main mineral component of bone and dental tissue, so the first biocompatible ceramics consisted of synthetic hydroxyapatite Ca_10_(PO_4_)_6_(OH)_2_ (HAp) and afterwards β-tricalcium phosphate, β-Ca_3_(PO_4_)_2_ (β-TCP), including composites based on them (HAp/TCP) in a ratio from 4:6 to 6:4 [15]. Bioresorption of HAp and TCP is a crucial factor in bone formation that should be optimized. One of the ways to increase bioresorption is to add sodium ions to the composition of the material. Double calcium phosphates have a higher solubility relative to TCP and, accordingly, HAp, due to the replacement of the Ca^2+^ ion by an ion with a larger radius and (or) a lower charge [16].

Sodium ions provide an antibacterial effect and promote early dissolution of implant material with the formation of layers rich in hydroxyapatite (HAp) and hydroxyl carbonate apatite (HCA); are associated with membrane functions (conducting nerve impulses, maintaining electrical potential on the membrane, Na^+^, K^+^ pump operation, maintaining anionic, cationic, and osmotic balance); and help regulate water balance in the body [13,16].

Oxides of sodium, calcium, silicon, and phosphorus form the Na_2_O–CaO–SiO_2_–P_2_O_5_ system, of which the most famous material—Bioglass 45S5—was developed in 1969 by Professor Larry Hench [17,18,19]. However, this system is not limited to the variety of glasses and glass–ceramics derived from the 45S5 bioglass concept. The system is sufficiently extensive to open the possibility of obtaining composite materials based on bioglass and calcium phosphate materials [20,21]; ceramics from powders produced by mixed-anion synthesis [22]; and ceramic materials using intermediate phases, which can be converted into final compounds during heat treatment [23]. This review aims to consider approaches to obtaining such materials and their properties.

## 2. Bioglass 45S5

### 2.1. The Na_2_O–CaO–SiO_2_–P_2_O_5_ System

**Figure 1:**
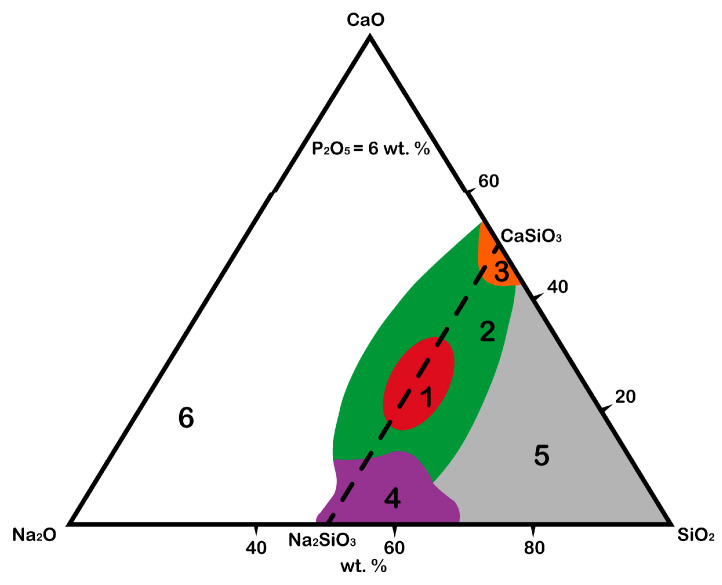
Schematic of bioactive regions in the Na_2_O–CaO–SiO_2_ system with fixed amount of P_2_O_5_.

The original idea of Larry Hench was to combine the elements that a human body is replete with in proportions that facilitate the rapid release of alkalis from the glass surface in aqueous solutions, followed by the formation of layers rich in calcium and phosphorus. Thus, the Na_2_O–CaO–SiO_2_ system was chosen as a base, to which phosphorus oxide (P_2_O_5_) was also added in small amounts (6 wt.%) since it was believed that its presence in glass or ceramics was critical to the bioactivity of the material at the time [24]. However, the development of modern approaches to obtaining an “ideal” biomaterial has shown that phosphate-free glasses and ceramics also have bioactivity [25,26]. For instance, calcium silicate (CaSiO_3_) bioceramics have significantly greater osteoinductive capacity, and it has been observed both in vitro and in vivo compared with tricalcium phosphate [25]. Calcium silicate extract promoted macrophage polarization, thus reducing the host-to-material inflammatory response. In addition, after stimulation by a macrophage-conditioned medium pretreated by calcium silicate extracts, the osteogenic differentiation of bone marrow stromal cells (BMSCs) was greatly enhanced by macrophage-derived oncostatin M [25]. Nevertheless, phosphorus oxide plays a specific role in the composition of the material (it accelerates the formation of hydroxyapatite and hydroxyl carbonate apatite layers on the surface of the material), so its presence is desirable in the final product. Thus, the Na_2_O–CaO–SiO_2_–P_2_O_5_ system can be schematically presented in a Na_2_O–CaO–SiO_2_ phase diagram (Figure 1) with the fixed mass value of P_2_O_5_ equal to 6 wt.% [17].

The diagram shows the areas responsible for the possibility of the formation of chemical bonds between material and native bone tissue after the integration of the material into a body [19,27]:

- The red area (1) is the area of biological activity of class A, in which the corresponding biologically active glasses are osteoproductive (bind to bone and soft tissue, activate genes). The formation of the HAp layer is observed several hours after integration into a body.
- It was proved that bioglass grade 45S5 (Bioglass^®^) forms such a strong bond with a bone that the implant cannot be removed without destroying it [28]. This effect is observed due to the saturation of the bone tissue defect with calcium and silicon ions, which stimulate osteogenic cells to form a bone matrix;
- The green area (2) is the area of biological activity of class B, in which the corresponding biologically active glasses are osteoconductive (bind only to bone tissue). The formation of the HAp layer is observed from 24 to 96 h after integration into a body;
- The orange area (3) is the area of biological activity in which the formation of Cerabone bioglass ceramics takes place, consisting of apatite (Ca_10_(PO_4_)_6_(OH_1_F_2_)) and wollastonite (CaO·SiO_2_) crystals as well as a residual SiO_2_ glassy matrix [19]; however, unlike in other areas, the P_2_O_5_ content may vary;
- The purple area (4) is where the corresponding biologically active glasses are fully resorbed in the body after 10 to 30 days with a minimal restoration of damaged bone tissue;
- The gray area (5) is where the corresponding glasses do not form bonds even with bone and behave like a bioinert material;
- The white area (6) is where glass formation is not observed.

Traditionally, the Na_2_O–CaO–SiO_2_ system has been used in the production technology of soda lime silicate glasses (windows, glass, bottles, containers, and so on) where Na_2_O, CaO, and SiO_2_ are the main components. The differences between bioactive glasses and industrial glasses are [19]: (1) SiO_2_ content less than 60 mol%, (2) high Na_2_O and high CaO content, and (3) high CaO/P_2_O_5_ ratio. These compositional features make the surface highly reactive when it is exposed to an aqueous medium.

#### 2.1.1. Benefits and Drawbacks of Bioactive Glass 45S5

In the middle of the 1980s, Bioglass 45S5 was introduced to the market, and it stimulated many research groups to an intensive investigation of bioactive glasses and their further applications. Today, more than 1000 articles have been published [24] dedicated to studying the physicochemical properties of Bioglass 45S5 and resembling materials, its bioactivity, bioresorbability, and the limits of its applicability. A massive array of data makes it possible to highlight both the benefits and drawbacks of bioglass compositions in the Na_2_O–CaO–SiO_2_–P_2_O_5_ system.

The main benefit of bioglass materials in the Na_2_O–CaO–SiO_2_–P_2_O_5_ system is that their dissolution products enhance the expression of genes that control osteogenesis [29,30]; thus, they provide a higher rate of bone formation in comparison with other inorganic bioceramic materials, such as hydroxyapatite [31]. One of the explanations is the presence in most bioglasses of sodium oxide (Na_2_O) in sufficiently large quantities to create an alkaline environment, providing an antibacterial effect and promoting early dissolution of the implant material with the formation of layers rich in HAp, HCA, and silicon dioxide [18,32]. The studies [33,34] demonstrated that Bioglass 45S5 is efficient against Staphylococcus aureus, Staphylococcus epidermidis, and Escherichia coli.

The drawbacks of bioglass materials in the Na_2_O–CaO–SiO_2_–P_2_O_5_ system are again associated with a high alkali content, namely: a relatively fast rate of dissolution and resorption [24,35], which negatively affects the balance of natural remodeling of bone tissue and leads to the formation of a gap between the tissue and the implant material [36]; low mechanical strength of scaffold structures; and significant cytotoxic effect caused by high doses of sodium leached into the culture medium [37].

One of the currently used methods for increasing the mechanical strength of bioglass materials is devitrification, as a result of which crystalline phases are formed in the material.

#### 2.1.2. Methods for Obtaining Crystal Phases

Devitrification of bioglass is a simple method for obtaining crystal phases and includes glass preparation, grinding obtained glass to a size of 60–150 microns, and the firing of glass powder at temperatures no higher than 1050 °C with an exposure of 1.5–2 h. This approach is not universal and has a generalized character according to the literature data. Additional components can be added to a glass charge to form crystal phases of a given composition while an individual heat treatment mode is selected.

Bioactive glasses can be obtained by two methods (Table 1): traditional quenching from a melt and sol–gel.

Nevertheless, the approach to obtaining crystal phases has difficulties related to forming stoichiometric compounds and repeatability limits. The phase compound of such materials can either coincide or differ from the composition of the initial amorphous phase [46,47]. Thus, the formation of both one phase and many crystalline phases of different chemical compositions with preservation of the bioactivity of some can occur during devitrification.

On the other hand, spark plasma sintering (SPS) may be used for producing fully dense and completely amorphous Bioglass 45S5 specimens at temperatures as low as 500–550 °C and crystallized Bioglass 45S5 specimens with Na_2_CaSi_2_O_6_ phase composition at 600 °C. It has been confirmed the even higher bioactivity of Na_2_CaSi_2_O_6_ is obtained by SPS compared to by amorphous Bioglass 45S5 also produced by SPS at 500–550 °C or conventionally sintered Bioglass 45S5 (crystallized by high-temperature treatment at 1050 °C) [48].

### 2.2. The Na_2_SiO_3_–CaSiO_3_ System

**Figure 2:**
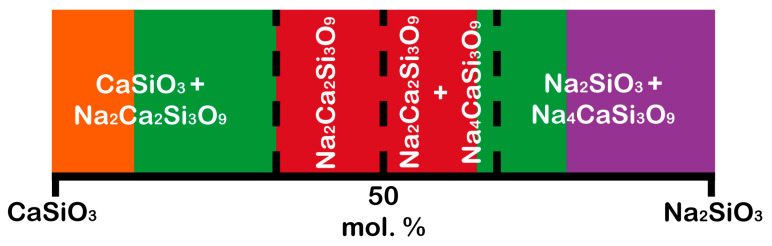
Schematic of bioactive regions in the Na_2_SiO_3_–CaSiO_3_ system. The red color is the area of biological activity of class A; the green color is the area of biological activity of class B; the orange color is the area of obtaining the Cerabone bioglass ceramics; the purple color is bioglasses provide a minimal restoration of damaged bone tissue due to their high resorption. (Color description is related to Figure 1).

The area of biological activity of classes A and B, where the corresponding biologically active glasses are osteoproductive and osteoconductive, respectively, is located on the line of the Na_2_SiO_3_–CaSiO_3_ phases (Figure 1). Therefore, it can be assumed that the crystallization of the compounds lying on the line of these phases can form biologically active crystalline phases (Figure 2).

The pseudo-binary Na_2_SiO_3_–CaSiO_3_ system includes five crystalline phases [49]: CaSiO_3_ (wollastonite (β-CaSiO_3_), JCPDS 43-1460, and pseudowollastonite (α-CaSiO_3_), JCPDS 31-300), Na_2_O·CaO·2SiO_2_ (112, JCPDS 77-2189), Na_2_O·2CaO·3SiO_2_ (123, combeite, JCPDS 75-1686), 2Na_2_O·CaO·3SiO_2_ (213, JCPDS 37-0282), and Na_2_SiO_3_ (JCPDS 16-0818).

Comparison between glasses (CaSiO_3_, 112, 123, 213, and Na_2_SiO_3_) and crystalline phases (CaSiO_3_, 112, 123, 213, and Na_2_SiO_3_) demonstrated that crystalline phases did not inhibit the development of the HAp and HCA layers due to P_2_O_5_-free compositions possessing the ability to form these layers because phosphorus ions P^5+^ can be obtained from human blood. Nevertheless, crystalline phases affected their formation rate [32]. In other words, the formation of the HAp and HCA layers on the crystalline phases was observed to be 2–4 times slower than on the corresponding glasses while maintaining bioactivity [47,50]. Thus, by selecting the composition of a biomaterial, it is possible to control the rate of its resorption in the body.

Speaking of calcium silicate (CaSiO_3_) ceramics, it is worth noting that CaSiO_3_ has excellent biocompatibility and mechanical properties compared to hydroxyapatite, as well as high bioactivity and a faster dissolution rate compared with calcium phosphate materials [51,52,53]. In addition, a relatively extensive chemical composition range of silicate ceramics allows for optimization of the phase composition to improve the mechanical properties of bioactive ceramics in contrast to phosphate ceramics [54].

The addition of sodium silicate leads to sodium–calcium silicates formation, which has been studied in works [55,56,57]. For instance, Durgalakshmi et al. [55] estimated the biocompatibility of bioglass with phase composition Na_4_CaSi_3_O_9_ and Na_2_Ca_3_Si_6_O_16_ from MTT assay, where the absorbance of the MTT assay for 3T3 cell lines was observed at 570 nm for all the samples after three days. The result showed that all materials had 50% to 60% cell viability for fibroblast cell lines concerning osteoblast cell lines in bone reconstruction (the percentage of fibroblast formation must be less but not zero). The authors demonstrated that crystallized bioglass with phase composition Na_4_CaSi_3_O_9_ and Na_2_Ca_3_Si_6_O_16_ is a prospect in tissue engineering applications.

Moreover, Lin et al. [56] noted several aspects, for example, the comparable elastic moduli of the 45S5-derived crystal phase (Na_2_CaSi_2_O_6_) with hydroxyapatite and the ability to develop an HCA layer in simulated body fluid (SBF); in comparison with the Na_2_CaSi_2_O_6_, 45S5 glass possessed a higher in vivo bioactivity index due to it being amorphous. It indicates that the 45S5-derived crystal phase (Na_2_CaSi_2_O_6_) is better suited for use as a substitute for bone than its glass.

In addition, work [57] demonstrated the bioactivity of Na_2_Ca_2_Si_3_O_9_ by soaking Na_2_Ca_2_Si_3_O_9_ disks in SBF. The results in vitro showed that hydroxyapatite (HAp) formed on the surface of Na_2_Ca_2_Si_3_O_9_ samples after soaking for 1 day, which indicated the good bioactivity of Na_2_Ca_2_Si_3_O_9_.

According to the literature data, the main crystal phase formed during the devitrification of pure Bioglass 45S5 glasses has not been established precisely. Some authors indicated the formation of the Na_2_Ca_2_Si_3_O_9_ phase [58,59,60]; others pointed at the Na_6_Ca_3_Si_6_O_18_ phase formation [44,46]; and there are works in which Na_4_Ca_4_Si_6_O_18_ was determined as the crystalline phase after glass devitrification [23,61]. The current uncertainty can be explained by the close lattice parameters of all three compounds, Na_2_Ca_2_Si_3_O_9_ (hexagonal, P31 (152), a = 10.464, c = 13.176) [62], Na_4_Ca_4_Si_6_O_18_ (hexagonal, P32 (152), a = 10.464, c = 13.168) [63], and Na_6_Ca_3_Si_6_O_18_ (rhombohedral, R-3m (166), a = 10.500, c = 13.184) [64], as well as by the isostructurality of the Na_6_Ca_3_Si_6_O_18_ and Na_4_Ca_4_Si_6_O_18_ phases and the high-temperature form Na_2_Ca_2_Si_3_O_9_. Thus, clear identification of the main crystalline phase of ceramic from sintered bioglass is impossible due to the close structural similarity of these phases. Nevertheless, work is underway to study crystal phases in glass–ceramics. For example, the authors of [46] gave several arguments in favor of the Na_6_Ca_3_Si_6_O_18_ phase formation after the devitrification of Bioglass 45S5. Sodium–calcium silicate phase determination is a challenging task, and it becomes more sophisticated when composites are obtained.

## 3. Calcium Phosphate/Bioglass 45S5 Composites

Calcium phosphates are unique materials in which the resorption rate depends on the Ca/P ratio. The lower the Ca/P ratio is, the more resorption of calcium phosphate materials occurs. Hence, hydroxyapatite (HAp, Ca_10_(PO_4_)_6_(OH)_2_, Ca/P = 1.67) is thermodynamically stable at physiological pH and body temperature; tricalcium phosphate (TCP, Ca_3_(PO_4_)_2_, Ca/P = 1.5) resorbs rapidly in vivo (α-TCP has a higher dissolution rate then β-TCP in the body); and calcium pyrophosphate (CPP, Ca_2_P_2_O_7_, Ca/P = 1) and dicalcium phosphates (brushite or DCPD (CaHPO_4_·2H_2_O, Ca/P = 1) and monetite or DCPA (CaHPO_4_, Ca/P = 1)) resorb harmfully fast [65]. The Bioglass 45S5 addition to calcium phosphates promotes composites which possessed unique physicomechanical properties and promising results in the in vitro and in vivo testing discussed below.

### 3.1. Hydroxyapatite/Bioglass 45S5 Composites

Hydroxyapatite is a material of bones and teeth, has high biocompatibility and osteoconduction properties, and belongs to the second generation of biomaterials [66,67], as does Bioglass 45S5 [68]. Amorphous and natural HAp has the ability to guide the growth of new bone and forms chemical bonds with bone tissue after implantation (no formation of fibrous capsule), due to which the bonding strength increases compared to the bond strength between bioinert ceramics and bone [69]. This effect can be explained by a relatively hydrophilic surface: by the presence of hydroxyl groups in HAp, which remain stable in the HAp structure up to 1350 °C and strongly interact with polar chemical compounds [70]. Hence, hydroxyl groups have a high affinity for amino acids, proteins, and organic acids in the human body through hydrogen bonds [71]. However, synthetic high-temperature HAp has relatively high stability in terms of temperature, pH, and composition of intravascular fluid, as well as high crystallinity; hence, all of these properties make it difficult to degrade in the human body [66,72,73,74].

The hydroxyapatite/Bioglass 45S5 composites are prospective composites for the regeneration and repair of bone tissue applications. Much research has been carried out with different approaches to the combination HAp and bioactive glass 45S5 for the investigation of the properties of obtained composites and coatings: spark plasma sintering (SPS) [75,76,77], pressureless sintering [20,78,79,80,81,82,83], electrophoretic deposition (EPD) [84], electrospinning [85], pulsed laser deposition (PLD) [86,87], and CoBlast coating [88]. Cement composites of HAp with Bioglass 45S5 can also be obtained by the reaction of calcium phosphate precursors, where, for producing HAp, for instance, tetracalcium phosphate (Ca_4_(PO_4_)_2_O, TTCP) with dicalcium phosphate dihydrate (CaHPO_4_·2H_2_O, DCPD) or dicalcium phosphate anhydrite (CaHPO_4_, DCPA) is used [89,90,91].

The phase composition, mechanical properties, and bioactivity of composites depend on initial parameters: pressure, temperature, the concentration of bioglass, exposure time, and other parameters specific to each method (Table 2).

In fact, Demirkiran et al. [20] investigated sintering composites (100 − x)HAp/x45S5 (x = 1, 2.5, 5, 10, 25 wt.%) at 1200 °C for 4 h by XRD and XANES analysis. The authors demonstrated that the composites did not contain calcium silicates and sodium phosphate; nevertheless, the composites contained primary sodium silicates in the amorphous state and primary phosphate represented by hydroxyapatite or β-TCP. Composites with x = 1–5 wt.% showed that as the amount of 45S5 addition increased, the decomposition of hydroxyapatite to yield β-TCP increased (decomposition of hydroxyapatite in the presence of ≤5wt.% of 45S5 was triggered by the migration of Ca^2+^ ions out of the structure to form CaO that reacted with P_2_O_5_ contained in 45S5 and formed β-TCP) [82]. In contrast, Rizwan et al. [76,77] investigated composites of (100 − x)HAp/x45S5 (x = 0, 2.5, 5, 10, 15, 20, 25, 30 wt.%) obtained by spark plasma sintering. They demonstrated that the greater amount of 45S5, the more the transformation HAp to β-TCP decreased due to the stabilization of HAp by 45S5 (diffusion of Na and Si in the calcium phosphate phase was considered responsible for the stabilization of HAp with an increase in bioglass content, while diffusion of Ca from the calcium-phosphate-based phase made the bioglass less vulnerable to crystallization).

In the works discussed above, the composites showed excellent bioactivity in the in vitro tests. Thus, they illustrate the influence of the method used for HAp/Bioglass 45S5 composites obtained in the final phase composition; thereby the final material still possesses specific characteristics such as antibacterial effect, bioactivity, and mechanical properties which surpass the properties of the individual HAp and Bioglass 45S5. The biological performance of the final system can be controlled by modifying the volume or mass fractions of the constituents.

### 3.2. Tricalcium Phosphate/Bioglass 45S5 Composites

The rate of β-TCP resorption is much higher than for HAp [92], and its decomposition products provide Ca^2+^ and PO_4_^3-^ ion saturation for osteoblasts, causing relatively rapid bone regeneration [93]. β-TCP is obtained by solid phase reactions at temperatures above 800 ℃, and at temperatures above 1150–1200 °C, β-TCP is converted to α-TCP [94,95]. It is worth noting that β-TCP can form bonds directly with bone tissue after implantation into the body without the intervention of fibrous connective tissue [96]. Moreover, the addition of human-bone-marrow-derived mesenchymal stem cells (hBMSCs) to β-TCP improved the bone-forming capacity of such composites (hBMSCs/β-TCP) since immunohistochemical expression of human osteocalcin was detected from the seventh week [97], and the formation of lamellar bone tissue was observed in both subcutaneous tests and intramuscular constructs.

However, as HAp does, β-TCP exhibits poor mechanical properties which limit its applications as a loadbearing (3–5 MPa) biomaterial due to its poor fatigue resistance and brittleness [98,99]. In addition, the β-TCP degradation can be too fast to have enough available surface area for cell spreading and to match the growth rate of new tissue, which may lead to the degradation of the implant before the healing process is complete [98,99]. Hence, it may be one of the reasons to limit research on β-TCP/Bioglass 45S5 composites and focus more on improving the biological and mechanical performance of HAp/Bioglass 45S5 composites.

Despite the lack of research dedicated to β-TCP/Bioglass 45S5 composites, there have been some sufficiently interesting studies. For instance, Chitra et al. [100] compared the bioactivity of Bioglass 45S5 after sintering to β-TCP. The 45S5 phase composition after sintering at 600 °C included Na_2_Ca_2_Si_3_O_9_ and Na_2_Ca_3_Si_6_O_16_. The SBF test showed apatite layer formation on the first day for 45S5 and on the seventh day for β-TCP. The β-TCP showed elevated compatibility as compared to 45S5 by hemocompatibility assay (erythrocyte cells), but 45S5 showed a higher ratio of compatibility than the β-TCP by cytocompatibility assay (MG-63 cells). The authors noted that comparative analysis demonstrated the possibility of producing better bioactive/biocompatible and bioresorbable materials by preparing composites of 45S5/β-TCP with the maximum proportion of 45S5.

It is worth considering that β-TCP/45S5 biocomposites have potential for bone and dental tissue engineering, which was represented by Sujon et al. [21]. The authors investigated (100 − x)TCP/x45S5 (with x = 40 wt.%) with a powder composition based on sol–gel-derived 45S5 incorporated with β-TCP for potential application in dental tissue engineering. The Bioglass 45S5 obtained by sol–gel consisted of Na_2_Ca_2_Si_3_O_9_ after sintering at 600 °C for 1 h, so the TCP/45S5 powder included Na_2_Ca_2_Si_3_O_9_ and β-TCP. The average particle size of the powder was 32.4 µm. An SBF test showed a denser apatite layer formation for TCP/45S5 composition after 14 days of immersion compared to 45S5 and β-TCP. The Saos-2 cells were able to attach, spread, and proliferate safely without toxic interference from the composition.

Thus, β-TCP/Bioglass 45S5 composites have potential for application in bone and dental tissue engineering; however, an individual approach to each bone tissue defect will make it possible to expand the application boundaries of such composites. There have been several works in the last decade dedicated to β-TCP/Bioglass 45S5 composites [101,102,103,104,105,106,107,108,109] or investigation of biphasic calcium phosphate (BCP)/Bioglass 45S5 composites [110] obtained with the use of additive technologies (Table 3). The addition of HAp to β-TCP (biphasic calcium phosphate) aims to achieve simultaneously both controllable biodegradation and biological stability [98].

On the other hand, the phase composition of the β-TCP/45S5 composites is more predictable than for HAp/45S5 composites; it depends on the amount of Bioglass 45S5, temperature, and time of sintering. These parameters also have an influence on the bioactivity and mechanical properties of β-TCP/45S5 composites.

For instance, the authors of [105] noted that Bioglass 45S5 induced a phase transition from β-TCP to Si–TCP by replacement of phosphorus with silicon in the lattice (substitution reached saturation at 5–7.5 wt.% 45S5) at temperatures lower than the theoretical temperature due to Si possibly reducing the thermal stability of β-TCP to lower temperatures, enabling the Si/α-TCP phase type. In addition, Na’s presence in Bioglass 45S5 helps soften the TCP surface at lower temperatures than usual and acts as a glue to enhance densification and mechanical strength during sintering [105,106]. Liquid phase sintering helps crack bridging and crack deflection, leading to the advanced compressive strength of the composite [54,106]. Consequently, enhancement of Bioglass 45S5 content up to 7.5 wt.% leads to α-TCP or Si–TCP formation. Further increasing the Bioglass 45S5 content leads to reaction between TCP and 45S5 during sintering, producing CaSiO_3_, NaCaPO_4_, and Na_2_Ca_2_Si_3_O_9_ as the 45S5 content increases.

Moreover, the greater the exposure time or the Bioglass 45S5 content is, the more growth and roughening of ceramic grains are observed and the greater the possibility of cracks formation [109,110]. However, any comparison should be carried out for composites obtained with the same approach and initial parameters with a similar pore distribution.

In addition, the composition of the bioactive glass also plays a crucial role in the phase composition, bioactivity, and mechanical properties of composites [29,54,99].

Thus, β-TCP/45S5 composites are prospective objects with improved bioactive and mechanical properties due to the combined advantages of the β-TCP and the Bioglass 45S5.

### 3.3. Calcium Phosphate with Ca/P = 1/Bioglass 45S5 Composites

#### 3.3.1. Dicalcium Phosphates/Bioglass 45S5 Composites

Calcium phosphate cements (CPCs) are attractive biomaterials due to their ability to harden after in situ implantation or injection [111]. Especially, the workability of CPCs as injectable bone graft substitutes (IBS) extends their field of application and overcomes the barriers associated with repairing unevenly shaped bone defects with preformed scaffolds [111,112].

The advantages of CPCs include high biocompatibility and bioactivity, nontoxicity, an abundant source of calcium and phosphate ions, and the ability to easily hand-moulding of blocks with complex shapes. The disadvantages of CPCs include being hardly suited for critical bone defects, the lack of bone tissue integration to accelerate new bone formation, low mechanical properties and pH, the absence of macro-pores (only intrinsic nano-pores), and sometimes very high setting time [89,112,113,114].

The cement of the dicalcium phosphate dihydrate CaHPO_4_·2H_2_O (DCPD, or brushite) is obtained as a rule via the interaction of β-TCP and monocalcium phosphate monohydrate (MCPM) by Reaction (1) [23,115]:

Ca_3_(PO_4_)_2_ + Ca(H_2_PO_4_)_2_ H_2_O + 7H_2_O → 4CaHPO_4_ 2H_2_O, (1)

Brushite cement has weak and brittle mechanical properties, as well as an acidic nature, but a high rate of in vivo resorption and re-crystallization into an apatite, which is generally resorbed by osteoclasts; the dicalcium phosphate is resorbed by simple dissolution and more predominantly by cell mediation (early brushite resorption is regulated by macrophages) [23,99,111,116].

The presence of unreacted brushite cement after implantation may lead to its transformation to less soluble octacalcium phosphate or HAp phases, which are subsequently osteoclast mediated. Interestingly, monetite possessed higher resorption and bone formation in vivo than brushite, notwithstanding the uniform resorption mechanisms. It may be explained by the absence of phase conversion to apatite for monetite types of cement [116].

As in the case of all other calcium phosphates, the incorporation of Bioglass 45S5 helps to enhance bioactivity. It happens due to bioglass-deposited ions enhancing apatite aggregation on the cement surface, leading to host–bone interactions [112]. Bioactivity can be controlled by the Bioglass 45S5 content. Hence, CPC/Bioglass 45S5 composites are attractive and prospective objects of investigation due to their improved biocompatibility and mechanical properties.

In fact, Lukina et al. [113,117] investigated bioglass-powder-incorporating brushite cement composites (100 − x)brushite/xBioglass (x = 0, 10, 20 wt.%), where the composition of bioglass was close to that of Bioglass 45S5, i.e., 25 Na_2_O, 20 CaO, 50 SiO_2_, and 5 P_2_O_5_ wt.%. Brushite was obtained by Reaction (1). DCPD crystallizes in lamellar crystal forms (≈8 μm long and 2 μm wide), whose physical coupling and intertwining give the cement stone strength [117]. Brushite cement possesses porosity (≈40–45%), pH (≈3.3) and compressive strength (15 MPa), thereby 90brushite/10Bioglass composites demonstrated slightly enhanced pH and compressive strength up to ≈ 3.8 and 16 MPa, respectively, as well as a bimodal pore structure on holding in buffer solution [117]. Bioglass incorporation always enhances the pH as bioglass content increases, especially in composites containing crystallized glass granules compared to amorphous glass granules, but decreases the rate of brushite crystallization [113]. It was established that the optimal incorporated bioglass content should be not greater than 10 wt.%, irrespective of the form of the initial β-TCP and the type of glass (amorphous or crystalline), as such composites possess desirable properties [113]: strength in compression 12–20 MPa, pH 4.2–5.5, and high resorption rate 8–20%. The biocompatibility and bioactivity of these composites have been confirmed in vitro and in vivo [113,117].

On the other hand, Hasan et al. [112] investigated Bioglass-45S5-crystallized-powder-incorporated brushite cement composites (100 − x)brushite/x45S5 (x = 0, 10, 20, 30, 40, 50 wt.%). Brushite was obtained by Reaction (1). The crystallized Bioglass 45S5 consisted of predominantly Na_2_Ca_2_Si_3_O_9_ and Na_2_Ca_4_(PO_4_)_2_SiO_4_, demonstrating that the MC3T3-E1 cells were able to attach, spread, and proliferate safely without toxic interference from the microspheres. It was established that Bioglass-45S5-crystallized-powder-incorporated brushite cement did not change the setting time range (5 to 12 min) or compressive strength (≈ 10 MPa) significantly when its content was enhanced to 40 wt.% but still had an impact on these parameters. The SBF test showed homogeneous apatite layer formation and a better degradation rate for composites containing 45S5 rather than pure brushite. The higher biocompatibility and bioactivity of 45S5-containing composites were confirmed in vitro—the composites demonstrated the improved attachment, proliferation, and differentiation properties of the MC3T3-E1 cells without toxic interference from the composites—and in vivo: the 45S5 microspheres produced space for fibrous tissue recruitment and bone formation and accelerated implant degradation.

Despite the positive results illustrated for brushite/Bioglass 45S5 composites, further studies are needed to develop injectable bone substitute composites with the ideal specified properties. So, some investigations were devoted to extensions of the CPC phase composition [118,119]. For instance, the authors of [118] established that CPC/Bioglass 45S5 composites, where CPC included CaCO_3_ and DCPD, showed improved setting time, injectability, and compressive strength, while the authors of [119] established that CPC/Bioglass 45S5 composites, where CPC included α -TCP, DCPA, and HAp, showed improved soft tissue response and higher bone formation in a femoral condyle defects in rats in vivo.

#### 3.3.2. Calcium Pyrophosphate/Bioglass 45S5 Composites

The calcium pyrophosphate (CPP, Ca_2_P_2_O_7_) ceramic is an attractive and prospective object for investigation due to the molar ratio Ca/P = 1 and values of pH close to neutral (pH ~7) during immersion in water [120], which were confirmed by dynamic studies in this direction [120,121,122,123,124,125,126,127].

The CPP has several polymorphs [70]: amorphous-CPP, which is formed at 240–450 °C; γ-CPP, which is formed at ≈ 530 °C; β-CPP, which is formed at 700–750 °C; and β-CPP, which is transformed into α-CPP at 1140–1179 °C. According to the literature, most research was dedicated to the investigation of amorphous-CPP [122,123], γ-CPP [121], and β-CPP [120,125,126], which are produced by thermal conversion of hydrated ortho- or pyrophosphate calcium [122,123], solid phase synthesis [128], and the firing of brushite cement stone/powders [120,127,129] or monetite [125].

Despite the few pieces of research dedicated to CPP, there are some sufficiently interesting studies among them. For instance, Safronova et al. [120] investigated the phase evolution of the composites (100 − x)β-Ca_2_P_2_O_7_/xβ-Ca(PO_3_)_2_ (x = 0, 5, 10 mol.%; Ca/P = 1, 0.975, 0.95) during sintering obtained from calcium lactate pentahydrate Ca(C_3_H_5_O_3_)_2_·5H_2_O and monocalcium phosphate monohydrate Ca(H_2_PO_4_)_2_·H_2_O by mechanical activation. It was established that the phase composition for all composites after heat treatment at 600 °C was presented by γ-Ca_2_P_2_O_7_, and the lower the molar ratio Ca/P, the smaller the dimensions of the particles. Further increasing the temperature up to 900–1100 °C produced liquid phase sintering due to the presence of calcium polyphosphate β-Ca(PO_3_)_2_ as the greater the β-Ca(PO_3_)_2_ content and the temperature, the greater the effect of the liquid phase sintering. All composites fired at 900–1100 °C included the β-Ca_2_P_2_O_7_ phase. The biocompatibility was confirmed in vitro, so the MTT assay showed the viability assay of NCTC L929 cells in the presence of liquid extracts from ceramic samples, and the dental pulp stem cells (DPSC-32) were able to attach, spread, and proliferate safely without toxic interference from the β-Ca_2_P_2_O_7_ ceramics.

Moreover, Filippov et al. [127] investigated osteoconductive bioresorbable Ca_2_P_2_O_7_-based (100 − x)β-Ca_2_P_2_O_7_/xβ-Ca(PO_3_)_2_ (x = 10, 20, 30 wt.%) microporous ceramic scaffolds obtained by using the colloidal forming of reactive slurry into a plastic mold fabricated via fused deposition modeling 3D printing. It was established that the Ca_2_P_2_O_7_-based (x = 10 wt.%) composite ceramic, after sintering at 1000 °C for 1 h, included Ca_2_P_2_O_7_ and Ca(PO_3_)_2_ and possessed the highest compressive strength (54 MPa) and density (86%). Using this composition and conditions, the obtained ceramic scaffold with the Kelvin architecture possessed compressive strength and density equal to 1.4 MPa and 22%, respectively. The SBF test showed homogeneous apatite layer formation for all composites.

To the best of our knowledge, there have been no articles dedicated to research into the combinations of pure calcium pyrophosphate and Bioglass 45S5, or to their direct interactions during sintering. Nevertheless, the interactions between calcium pyrophosphate and Bioglass 45S5 may have been indirectly researched during the sintering of Bioglass-45S5-powder-incorporated brushite cement composites.

The following reactions were observed during heat treatment of the brushite [130]:

(2) $$2{\mathrm{CaHPO}}_{4}\;2H_{2}O\;\overset{200–450{}^{\circ}C}{\to }\;2{\mathrm{CaHPO}}_{4}+4H_{2}O,$$

(3) $$2{\mathrm{CaHPO}}_{4}\;\overset{500–650{}^{\circ}C5}{\to }\;\gamma \text{-}{\mathrm{Ca}}_{2}P_{2}O_{7}+H_{2}O,$$

(4) $$\gamma \text{-}{\mathrm{Ca}}_{2}P_{2}O_{7}\;\overset{700–1100{}^{\circ}C\;}{\to }\beta \text{-}{\mathrm{Ca}}_{2}P_{2}O_{7}$$

It is worth noting that Sventskaya et al. [23] investigated the porous 3D matrices of the composites (100 − x)brushite/x45S5 (x = 0, 25, 50, 75, 100 wt.%) for bone plastic surgery. It was established that heat treatment at 600 °C led to the formation of the β-Ca_2_P_2_O_7_, β-Ca_3_(PO_4_)_2_, and amorphous phases, while a further increase in temperature up to 700–900 °C led to obvious chemical reactions between calcium phosphates, especially β-Ca_2_P_2_O_7_, first of all, and Bioglass 45S5, which was characterized by the formation of NaCaPO_4_ and Na_4_Ca_4_(Si_6_O_18_), as Bioglass 45S5 content increased. The phase compositions for composites with x = 50 and 75 wt.% were presented by β-Ca_3_(PO_4_)_2_, NaCaPO_4_, and Na_4_Ca_4_(Si_6_O_18_) and predominantly Na_4_Ca_4_(Si_6_O_18_) after heat treatment at 900 °C, respectively. The compressive strength for these composites was at a maximum after sintering at 900 °C (6.1 and 5.6 MPa, respectively), while total porosity was the highest at 800 °C (49% and 42%, respectively), which was associated with the formation of plate-shaped crystals on the surface of the glass granules. The composites with x = 0 and 25 wt.% were characterized by non-uniform sintering, presence of cracks, and low compressive strength. The Bioglass 45S5 possessed the higher compressive strength, which increased exponentially from 600 °C (6.1 MPa) to 900 °C (34.6 MPa) due to full crystallization and formation of Na_4_Ca_4_(Si_6_O_18_) and CaSiO_3_ phases. It was established that the greater the 45S5 content in the composites, the more the pH of the contact medium increased, while the solubility of the composites decreased. Nevertheless, the authors demonstrated the approach of producing simple 3D matrices for bone plastic surgery using Bioglass 45S5 and brushite cement as precursors and the possibility of obtaining ceramic matrices with specified properties by regulating their ratio.

Calcium phosphates which react quickly in vivo may be less useful as bone fillers because of their excessively high dissolution rate as well as their weak and brittle mechanical properties [99,131]. This may explain the limited number of articles devoted to the study of calcium pyrophosphate compared to HAp or TCP and the cause of there being more limited articles dedicated to the investigation of CPP/Bioglass 45S5 composites correspondingly (to the best of our knowledge). On the other hand, it does not preclude the possibility of their production because such composites possess the synergistic combination of two bioactive phases; the biological response, the densification of the composite, and its mechanical strength will be improved [99]. The pyrophosphate ceramics have the low diffusion mobility of large pyrophosphate anions during sintering, which leads to a prevalence of re-crystallization phenomena over densification, so the resulting ceramics have low density and large grain size. This problem can be solved by liquid phase sintering, which assumes the presence of the component that ensures the formation of a liquid phase [127]. Bioglass 45S5 can fit this case; therefore, CPP/Bioglass 45S5 composites are interesting and prospective objects for future investigation.

## 4. Other Ways to Obtain Composite Ceramics in the Na_2_O–CaO–SiO_2_–P_2_O_5_ System

The ceramics or glass–ceramics in the Na_2_O–CaO–SiO_2_–P_2_O_5_ system may be obtained beyond the traditional approach (glass crystallization) in several other interesting ways: (1) through cement-like composites based on calcium phosphate (CP) powders (a filler) and an aqueous solution of sodium silicate (a binder) [16,132]; (2) through solid-state or liquid phase sintering of powders synthesized from mixed-anionic solutions [22]; (3) through cementation reaction during, for instance, 3D printing [107]; (4) through intermediate phases precipitated from the solution, which convert into final phases during the sintering [23]; or (5) due to the combination of these different ways.

Some of these approaches have already been considered in the current manuscript, so let us focus on the first two.

Kaimonov et al. investigated a novel approach to obtaining composite bioceramics based on HAp [132] or β-TCP [16] powders and an aqueous solution of sodium silicate (SS_aq_). It was established that the CP/SS_aq_ pastes possessed gradual hardening and strength gain (in several stages) depending on time and temperature; the plastic molding process was possible in the first 15 min of setting time (at the first stage). The hardening process depended on the amount and particle size of the filler, the silicate modulus of SS_aq_, and the humidity of the air, and it started at the paste’s surface–air interface, moving deep inside the material. The CP/SS_aq_ pastes demonstrated good adhesion and cohesion between the layers, as well as shape retention, in the model extrusion experiments. It is worth noting the absence of interaction between CP and SS_aq_ occurring after mixing, molding, solidification, and drying of the specimens; nevertheless, CP particles incorporated into SS_aq_ were bound to each other by amorphous, hydrated sodium silicate. It was established that the sintered ceramic samples experienced chemical interactions between CP and SSaq already at 500 °C with the formation of new phases; the phase composition was presented by Ca_10_(PO_4_)_6_(OH)_2_, Na_2_Ca_4_(PO_4_)_2_SiO_4_, and β-NaCaPO_4_ for HAp filler [132] and by β-Ca_3_(PO_4_)_2_, β-NaCaPO_4_, and SiO_2_ (cristobalite) for TCP filler [16]. Further, an increase in temperature up to 1100 °C in increments of 200 °C showed the phase evolution of the ceramic specimens; the phase composition at 1100 °C was presented predominantly by β-NaCaPO_4_, Na_2_Ca_3_Si_6_O_16_, and SiO_2_ (cristobalite) and a little by β-CaSiO_3_ for HAp filler [132] and predominantly by β-Ca_3_(PO_4_)_2_, Na_3_Ca_6_(PO_4_)_5_, and SiO_2_ (cristobalite) and a little by β-CaSiO_3_ for TCP filler [16]. The compressive strength increased exponentially from 7.2 MPa at 500 °C to 31.6 MPa at 1100 °C for HAp filler [132], while, for TCP filler, the compressive strength was already 31.1 MPa at 500 °C, and it was slightly enhanced up to 43.5 MPa at 1100 °C [16].

Furthermore, Golubchikov et al. [22] investigated a novel approach to obtain active powders by synthesis from mixed-anionic aqueous solutions for bioceramics production in the Na_2_O–CaO–SiO_2_–P_2_O_5_ system. The three types of powders, named as CaPSi, CaP, and CaSi, were prepared using a synthesis from aqueous solutions of Ca(NO_3_)_2_ and a mixed-anion solution containing Na_2_HPO_4_ and Na_2_SiO_3_, as well as solutions containing these sodium salts separately. It was established that the phase composition of the synthesized powders was presented by brushite for CaP powder, by calcium silicate hydrate for CaSi powder, and by calcium silicate hydrate and amorphous calcium phosphate for CaPSi powder. All powders included the reaction by-product of sodium nitrate (NaNO_3_), which played the role of a sintering aid and participant in the heterophase reactions of the formation of the ceramics during sintering. The reactivity of CaP powder was higher than that of CaSi powder. The main phases obtained for the ceramics based on the CaPSi powder were β-NaCaPO_4_, α-CaSiO_3_, and Na_3_Ca_6_(PO_4_)_5_, while, for the CaP and CaSi powders, the main phases were β-Ca_2_P_2_O_7_ and β-NaCaPO_4_ and α-CaSiO_3_ and Na_2_Ca_2_Si_2_O_7_, respectively.

To sum up, these works demonstrated alternative and prospective methods for obtaining bioceramics in the Na_2_O–CaO–SiO_2_–P_2_O_5_ system using, in the former case, cement-like compositions based on an aqueous solution of sodium silicate and calcium phosphate filler and, in the second case, powders synthesized from mixed-anionic solutions.

## 5. Conclusions

The current work illustrates a perspective on calcium phosphate/Bioglass 45S5 composites as they have better bioactivity and mechanical properties than the individual calcium phosphate materials or Bioglass 45S5. It will be interesting to compare composites obtained under the same conditions of hydroxyapatite, tricalcium phosphate, calcium pyrophosphate, and dicalcium phosphate with a fixed value of Bioglass 45S5 in a work in the future. Such works will allow the correct comparison of the bioactivity and mechanical properties of composites.

On the other hand, the composition of bioglass does not limit the Na_2_O, CaO, SiO_2_, and P_2_O_5_ oxides, so it is pretty flexible, as evidenced by the numerous works devoted to the study of both various bioglasses as implants and calcium phosphate composites based on them. Such variation is a crucial factor in obtaining idealized functional implants. However, this review was devoted solely to Bioglass 45S5 composition.

It is worth noting that the method of obtaining bioactive composites plays a crucial role and directly influences the final properties of the implants, as well as the area of application. Despite a lot of work related to the investigation of different biomaterials and commercial implants used in bone tissue engineering, a lot of work still exists in this field.

## Figures and Tables

**Table 1:** Methods for obtaining bioglass in the Na_2_O–CaO–SiO_2_–P_2_O_5_ system.

	Melt–Quench [38,39,40,41,42]	Sol–Gel [43,44,45]
Initial reagents	Sodium carbonate (Na_2_CO_3_); calcium carbonate (CaCO_3_) or calcium oxide (CaO); phosphorus pentoxide (P_2_O_5_); silicon dioxide (SiO_2_).	Tetraethylorthosilicate (TEOS) or tetramethylortosilicate (TMOS); triethyl phosphate (TEP); calcium nitrate tetrahydrate (Ca(NO_3_)_2_·4H_2_O); sodium nitrate (NaNO_3_).
Steps	Mixing of a charge in a mill for 2 to 5 h; Filling into a rhodium or platinum crucible of homogenized and mechanically activated powder; Firing at 1350–1600 °C for 2–6 h (preliminary thermal heating can be carried out at 900–1000 °C for 1.5–2 h); Pouring melt onto a cold steel plate (or distilled water) and air cooling until room temperature (or dried in furnaces at a temperature of 70–90 °C for 5–6 h); Annealing of glass at 480–500 °C for 2–4 h.	Mixing solutions with constant stirring for a long time (6–15 h); Hydrolysis and polycondensation (a catalyst (acid or alkali) is used, affecting the gel formation structure and particle size. When using an alkali, large clusters are formed, while, when using acids, spatial networks are formed); Sol formation and further sol-to-gel transition; Drying at 25–150 °C or cryo-drying; Sintering (annealing) to produce glasses or crystal structures.
Advantages	Traditional approach; simple.	Higher purity and homogeneity; a wide range of compositions; possibility of obtaining silica gel at room temperature.
Disadvantages	Energy intensive; requires complete homogenization of the melt; may lead to contamination from the chemical substances; no possibility to fabricate porous scaffolds.	Dependence on pH; monoliths of bioactive glass (d > 1 cm) have cracks due to the shrinkage that occurs during drying and the evaporation of the liquid by-products of the condensation reaction; alkoxides are not suitable for large-scale production.

**Table 2:** Characteristics of hydroxyapatite/Bioglass 45S5 composites and coatings in the Na_2_O–CaO–SiO_2_–P_2_O_5_ system.

	Composition	Parameters of Sintering/Coating/ Cementation	Brief Characteristics of Materials	Evaluation of the Biocompatibility/ Bioactivity of Materials	Ref.
**Composites**					
**Spark Plasma Sintering**	HAp/45S5 laminated structure (m(HAp)/m(45S5) = 2/1)	T = 1223 K, P = 40 MPa, t = 16 min	Sintering of 45S5 with HAp and counter-diffusion of Ca and Na ions were observed at the phase interface. Large HAp grains (2 μm) were formed in the diffusion area. Crystallization of the 45S5 did not occur. The strain and stress of HAp/45S5 ceramics were both increased by 19 times (5.9%) and 1.89 times (79.8 MPa) compared with HAp ceramics (0.31% and 42.2 MPa).	No measurements were taken	[75]
	(100 − x)HAp/x45S5, x = 0, 2.5, 5, 10, 15, 20, 25, 30 wt.% [76] (100 − x)HAp/x45S5, x = 0, 10, 20, 30 wt.% [77]	T = 1000 °C, P = 3.67 MPa, t = 30 min	The crystalline phases were absent from the chemical reactions between the constituents or from the devitrification of glass. HAp without 45S5 transformed to β-tricalcium phosphate (β-TCP). Crystallization of the 45S5 did not occur. The greater the amount of 45S5, the more the transformation of HAp to β-TCP decreased due to the stabilization of HAp by 45S5.	SBF (14 days of immersion) test showed apatite layer formation for all composites. Composites showed an inhibition property against all the Staphylococcus spp. compared with HAp. The hBMSCs were able to attach, spread, and proliferate safely without toxic interference from the scaffolds, especially on HAp/45S5 (x = 30 wt.%). The mineralization activity of the hBMSCs increased with an increase in 45S5 concentration. Enhanced BMP-2, COL-1 secretion, and the osterix intracellular expression were observed in the hBMSCs seeded on HAp/45S5 (x = 30 wt.%). Composites exhibited resistance to monocyte migration.	[76,77]
**Pressureless sintering**	(100 − x)CF/x45S5 (CF—cuttlefish bone powder), (100 − x)HAp/x45S5, x = 30 wt.%	T = 900 °C, heating rate = 10 °C/min, cooled rate = 10 °C/min, t = 3 h	The CF/45S5 composite after sintering included HAp, Na_3_Ca_6_(PO_4_)_5_, and β-TCP. There was no evidence of any crystalline silicate phases. Silicon in amorphous form was localized in Ca-deficient areas of the surface. The HAp/45S5 composite after sintering included HAp and Na_2_Ca_2_Si_2_O_7_. Both strain and stress of CF/45S5 and HAp/45S5 composites were increased compared with HAp ceramics.	Incubation in DMEM for 7 days showed apatite layer formation for all composites. The MG-63 cells were able to attach, spread, and proliferate safely without toxic interference from the scaffolds, especially on CF/45S5 composites. The CF/45S5 composite was more effective at promoting ALP production compared to HAp/45S5 (the composite showed up-regulation of ALP activity as early as 3 days after the seeding).	[78]
	(100 − x)HAp/x45S5, x = 0, 20, 40, 60, 80 wt.%	T = 800 °C, t = 3 h	The HAp/45S5 (x = 20, 40 wt.%) composites after sintering included HAp, Ca_5_(PO_4_)_2_SiO_4_, and β-TCP. The HAp/45S5 (x = 60, 80 wt.%) composites after sintering included Ca_5_(PO_4_)_2_SiO_4_ and NaCaPO_4_. The greater amount of 45S5 was, the composites’ density and molar volume were decreased. Opposite, the hardness of composites increased as 45S5 increased and had a maximum value at 60 wt.% of 45S5. However, the hardness of all HAp/45S5 composites was lower compared with pure HAp.	No measurements were taken.	[79]
	(100 − x)HAp/xBG_Ca (BG_Ca = 47.3 SiO_2_/45.6 CaO/4.6 Na_2_O/2.6 P_2_O_5_, mol.%); (100 − x)HAp/x45S5, x = 20, 40 wt.%	T = 800 °C (HAp/BG_Ca), T = 1150 °C (HAp/45S5), heating rate = 10 °C/min, t = 3 h	The HAp/BG_Ca composites included HAp and amorphous phase after sintering at 800 °C. There was no reaction between HAp and BG_Ca glass in it. The HAp/45S5 composites included CaSiO_3_ and NaCaPO_4_ after sintering at 1150 °C.	SBF (14 days of immersion) test showed apatite layer formation for all composites, especially for HAp/BG_Ca due to the amorphous phase presence. The MC3T3-E1 cells were able to attach, spread, and proliferate safely without toxic interference from the scaffolds, especially on HAp/BG_Ca composites. The HAp/BG_Ca with x = 40 wt.% showed a tendency to dramatically increase ALP activity in MC3T3-E1 between 2 and 7 days.	[80]
	(100 − x)HAp/xBioK (BioK = 46.1 SiO_2_/26.9 CaO/24.4 K_2_O/2.6 P_2_O_5_, mol.%); (100 − x)HAp/x45S5, x = 50 wt.%	T = 750 °C (HAp/BioK), T = 1150 °C (HAp/45S5), heating rate = 5 °C/min, t = 3 h	The HAp/BioK composite included HAp, amorphous phase, and KCaPO_4_ after sintering at 750 °C. There was no reaction between HAp and BioK glass in it. The HAp/45S5 composite included HAp, Na_2_Ca_2_Si_3_O_9_, Ca_3_(Si_3_O_9_), and NaCaPO_4_ after sintering at 1150 °C. Composites had a porosity of about 30 vol%. The initial HAp/BioK microhardness (≈ 60 HV) was lower than the HAp/45S5 (≈130 HV).	SBF (14 days of immersion) test showed apatite layer formation for all composites, but higher rate formation was obtained for HAp/BioK because HAp/45S5 has wide crystallization during the sintering process. The value of HAp/BioK microhardness remained almost stable in the SBF test, and it was in the same order as that of the HAp/45S5 after 14 days of immersion.	[81]
	(100 − x)HAp/x45S5, x = 1, 2.5, 5, 10, 25 wt.%	T = 1200 °C, heating rate = 4 °C/min, cooled rate = 10 °C/min, t = 4 h	Composites with x = 1–5 wt.% included HAp and β-TCP (conversion of HAp to β-TCP increased as the extent of 45S5 and had max (35%) at 5 wt.% of 45S5). There was no evidence of any crystalline silicate phases. These composites had closed porosity in the sintered product and higher levels of densification. Composites with x = 10 wt.% included Ca_5_(PO_4_)_2_SiO_4_, β-TCP, and amorphous phase. Composites with x = 25 wt.% included Na_3_Ca_6_(PO_4_, and amorphous phase. These composites had porosity (14 and 10%, respectively) and lower levels of densification. The compressive strength increased as 45S5 content increased.	Incubation in DMEM (with and without the BMSCs) for 6 days showed apatite layer formation for all composites, especially for 25 wt.% of 45S5. However, the DNA concentration was higher for composite with 10 wt.% of 45S5. The composite with 25 wt.% of 45S5 showed the highest level of ALP activity.	[82]
**Cements**					
**No sintering**	(100 − x)(TTCP+DCPA)/x45S5, x = 0, 10, 20 wt.%	100% relative humidity box, T = 37 °C, t = 24 h (a hardening liquid = potassium phosphate buffers)	The hardened CPC included only HAp, the CPC/45S5 with x = 10 wt.% included HAp and Ca_2_SiO_4_, and the CPC/45S5 with x = 20 wt.% included HAp, Ca_2_SiO_4_, and Ca_3_SiO_5_ after setting for 24 h in a 100% relative humidity box at 37 °C. The setting time of the cement pastes increased from 15 min to 21 and 25 min as 45S5 content increased. The injectability of pastes and compressive strength of hardened composites (after setting for 7 days) increased too. The compressive strength of the CPC/45S5 with x = 20 wt.% reached 26 MPa at 1 day and 40 MPa at 7 days compared with only 15 MPa and 22 MPa of CPC at the same time points, respectively.	SBF (14 days of immersion) test showed a homogeneous and dense apatite layer formation and a high degradation rate (which could be adjusted by controlling the 45S5 content) for 45S5-contained composites. The rat osteoblasts were able to attach, spread, and proliferate safely without toxic interference from the composites, especially on the CPC/45S5 with x = 20 wt.%. The ALP activity of cells cultured on this composite was significantly higher than that of those on the CPC. The 45S5-contained implants incorporated well with the surrounding tissue and exhibited more effective osteogenesis and osteointegration at the defect area than CPC with good biocompatibility and biodegradability in vivo (no inflammatory response, rejection, or necrosis).	[90]
	(100 − x)(TTCP+DCPA)/x45S5, x = 7.5, 15 wt.%	100% relative humidity box, T = 37 °C, t = 10 min (a hardening liquid = monosodium phosphate solution)	All composites included TTCP, nanomonetite, and amorphous 45S5 after setting for 10 min in a 100% relative humidity box at 37 °C. All composites included calcium-deficient hydroxyapatite, remaining TTCP, and amorphous 45S5 after hardening in SBF solution at 37 °C for 3 days. The composite with 15 wt.% included the remaining origin bioglass particles. The compressive strength decreased from 44 MPa to 30 MPa after hardening in SBF solution as 45S5 content increased, while setting time increased from 4 min to 11 min.	There was a rise in cytotoxicity of composite cements as 45S5 content increased. Nevertheless, composite with 7.5 wt.% of 45S5 stimulated the population growth of cells with culture time and the differentiation of MSC to osteoblast line even in the first two days after seeding.	[91]
**Coatings**					
**Electrospinning**	HAp/(45S5/PLGA)	t = 10, 20, 30 min (spinning time); rotated speed = 230 rpm (HAp scaffolds); pumping rate = 1 mL/h (45S5/PLGA); voltage = 13 kV	Scaffolds of HAp/(45S5/PLGA): 45S5/PLGA solution (45S5/PLGA = 1:10) was coated around the HAp scaffolds (replica method) through the electrospinning process. The greater the spinning time was, the more both the composite thickness and the amount of 45S5 increased. 45S5 was in a pure state in the composite fibers.	The MC3T3-E1 cells were able to attach, spread, and proliferate safely without toxic interference from the scaffolds. The scaffold with 30 min spinning showed a tendency to dramatically increase ALP activity and induced the protein expression of OPN in MC3T3-E1. The mineralization activity of the MC3T3-E1 was observed on day 21 for scaffolds with a greater 45S5 concentration.	[85]
**Pulsed laser deposition**	(100 − x)HAp/x45S5, x = 0, 5, 10, 20, 50, and 80 wt.%	T = 600 °C (substrate temperature) [86,87], T = 200 °C (substrate temperature) [86], heating rate = 40 °C/min, t = 2 h	All PLD films included HAp and β-TCP at 600 °C. There were no peaks corresponding to 45S5. The films possessed lower amounts of P and Na elements due to P_2_O_5_ and Na_2_O existing in 45S5 which escaped from the plume during laser deposition. For all films obtained at 600 °C, the film adhesion strength increased as 45S5 content increased up to 20 wt.% (max adhesion). The film adhesion strength slightly decreased between 20 and 50 wt.%, and over 50 wt.% it almost did not change. The deposited film was amorphous at 200 °C and had lower adhesion strength with the substrate than the crystalline film obtained at 600 °C [86].	The films ((100 − x)HAp/x45S5, x = 10, 20, 80 wt.% [87], and x = 50 wt.% [86]) were selected for biological safety evaluation and implantation experiment. The films did not cause a hemolytic reaction, and L929 mouse fibroblasts were able to proliferate safely without toxic interference from the films. The in vivo test indicated the implant deposited at 600 °C had higher bonding strength with the new bone tissue compared with the implant deposited at 200 °C [86]. The film with 20 wt.% of 45S5 deposited at 600 °C exhibited better osteoconduction but still not enough coupling between the implant and bone tissue under the load.	[86,87]
**CoBlast**	HAp/45S5	Pressure = 75 psi, nozzle angle = 82 and 78°, nozzle height = 8 and 16 mm, speed = 13 and 15 mm/s for HAp and 45S5	The HAp/455 coatings were compared with 45S5 coating and commercial HAp coating (OsteoZip). The average surface roughness between 45S5 and HAp/45S5 did not differ, but OsteoZip was slightly rougher than HAp/45S5. The tendency of hydrophilicity was: 45S5 > H Ap/45S5 > OsteoZip.	The tendency of protein adsorption and MG63 cell attachment was: 45S5 > HAp/45S5 > OsteoZip. The MG63 cells were able to attach, spread, and proliferate safely without toxic interference from the coatings. The tendency of osteocalcin expression was: HAp/45S5 > OsteoZip > 45S5. The HAp/45S5 coating had the best angiogenic potential.	[88]

***Abbreviations***: 45S5—Bioglass 45S5; ALP—alkaline phosphatase; BMP-2—bone morphogenetic protein 2; BMSCs—bone marrow mesenchymal stem cells; COL-1—collagen type 1; CPC—calcium phosphate cements; DCPA—dicalcium phosphate anhydrite; DMEM—Dulbecco’s Modified Eagle’s Medium; HAp—hydroxyapatite; MC3T3-E1—mouse-calvaria-derived pre-osteoblastic; MG-63—human osteosarcoma cells line; MSC—mesenchymal stem cell; OPN—osteopontin; PLD—pulsed laser deposition; PLGA—poly(lactic-co-glycolic acid); SBF—simulated body fluid; TCP—tricalcium phosphate; TTCP—tetracalcium phosphate.

**Table 3:** Characteristics of TCP/Bioglass 45S5 composites in the Na_2_O–CaO–SiO_2_–P_2_O_5_ system.

	Composition	Parameters of Sintering	Brief Characteristic of Materials	Evaluation of the Biocompatibility/ Bioactivity of Materials	Ref.
**Robocasting**	(100 − x)TCP/x45S5 x = 50 wt.%	T = 1150 °C, heating rate = 5 °C/min, t = 1 h	Composite β-TCP/45S5 after sintering included Na_4_Ca_4_Si_6_O_18_, Na_2_CaSiO_4_, and NaCaPO_4_. The scaffolds showed high resistance to applied load with average ultimate compressive strength of 17.39 MPa and did not break at once. The scaffold had a hierarchical and repeating ordered structure with an average pore diameter of about 580 × 470 µm and a total porosity of about 40.6%.	SBF (14 days of immersion) test showed apatite layer formation for the scaffold. The hADMSCs were able to attach, spread, and proliferate safely. Scaffold had acceptable cytocompatibility.	[101]
	(100 − x)TCP/x45S5, x = 0, 5, 20, 40, 60, 80, 100 vol.%	T = 1000 °C, T = 1100 °C, heating rate = 2 °C/min, cooled rate = 4 °C/min, t = 1 h	Composites after sintering at 1100 °C with x = 5 vol.% included β-TCP and HAp; with x = 20 vol.% included β-TCP, HAp, and β-CaSiO_3_; with x = 40, 60 vol.% included β-CaSiO_3_ and NaCaPO_4_; with x = 80 vol.% included NaCaPO_4_ and Na_2_Ca_2_Si_3_O_9_; with x = 100 vol.% included Na_2_Ca_2_Si_3_O_9_. Composites after sintering at 1000–1100 °C with x = 0–40 vol.% possessed porosity (hardly any sintering between the individual particles did not occur) and low bending strength (≤20 MPa), contrasting with x = 60 (≤20 MPa at 1000 °C and ≈45 MPa at 1100 °C) and 80 vol.% (≈105 MPa).	No measurements were taken.	[102]
**Foaming**	(100 − x)TCP/x45S5 x = 20 wt.% (Vitoss BA Foam Pack) x = 0 wt.% (Vitoss Foam Pack)	-	Cylindrically shaped scaffolds were obtained (height ≈ 2.7 mm, diameter = 5 mm). The total scaffold volume was 31.86 mm^3^ for Vitoss and 34.58 mm^3^ for Vitoss BA scaffolds.	The hBMSCs were able to attach, spread, proliferate, and differentiate safely without toxic interference from the scaffolds, especially on Vitoss BA. It was established that the hBMSC differentiation and maturation were faster in Vitoss BA (higher ALP and osteogenic target genes (COL-1A, SPP-1, RUNX-2) activity) due to Vitoss BA possessing higher pH. The viability of the cells increased significantly in Vitoss BA.	[103]
**Immersion**	The β-TCP scaffolds were immersed 3 times under pressure in the sol-gel solution of 45S5 (90%TCP/10%45S5).	T = 1200 °C, heating rate = 5 °C/min, t = 2 h	Composites of β-TCP/45S5 after sintering included β-TCP, α-TCP, SiO_2_, Na_15,78_Ca_3_(Si_6_O_12_), and CaSiO_3_. The density, porosity, and compressive strength of the β-TCP (0.8 g/cm^3^, 72.4%, 2.98 MPa) and β-TCP/45S5 (0.8 g/cm^3^, 72.9%, 3.29 MPa) scaffolds were similar.	SBF test showed apatite layer formation for β-TCP/45S5. The MG-63 cells were able to attach, spread, and proliferate safely without toxic interference from the scaffolds, especially on β-TCP/45S5. The ALP activity was slightly higher on the β-TCP/45S5 surface. β-TCP/45S5 inhibited the proliferation of *E. coli*, *S. aureus*, and *C. albicans*.	[104]
**Gel-casting**	(100 − x)TCP/x45S5 x = 5, 7.5 wt.%	T = 1200 °C, heating rate = 5 °C/min, t = 2 h	Scaffolds of β-TCP/45S5 possessed bimodal porosity similar to β-TCP scaffolds (≈83–84%), but their compressive strength was twice as high (≈1.5 MPa). Composites of β-TCP/45S5 after sintering included β-TCP and Si/α-TCP. Any crystalline phase related to the Bioglass 45S5 did not occur.	The MG-63 cells were able to attach, spread, and proliferate safely without toxic interference from the scaffolds, especially on (100 − x)TCP/x45S5 with x = 7.5 wt.%. This scaffold showed higher viability of the cell.	[105]
**Binder jetting**	(100 − x)TCP/x45S5 x = 5, 10 wt.%	T = 1250 °C, t = 2 h	Composites of β-TCP/45S5 after sintering included β-TCP, α-TCP, and Na_2_Ca_2_Si_3_O_9_. Among all scaffolds, the scaffolds with 5 wt.% of 45S5 possessed the highest bulk density (g/cm^3^) and compressive strength (MPa) but the lowest total porosity (%) for scaffolds with random (1.6 g/cm^3^, 26.7 MPa, 47.9%) and designed (1.4 g/cm^3^, 21.3 MPa, 54.1%) porosity, respectively.	SBF test showed apatite layer formation for all composites, especially those that were 45S5 contained. The MTT assay results with human osteoblast cells (hFOB) showed cells were able to attach, spread, and proliferate safely without toxic interference from the scaffolds.	[106]
	(100 − x)TCP/x45S5 x = 60 wt.%	T = 1000 °C	Composites of β-TCP/45S5 after sintering included NaCaPO_4_ and CaSiO_3_. Composites possessed a bending strength of ≈15 MPa after sintering at 1000 °C.	No measurements were taken.	[107]
**Sintering**	(100 − x)TCP/x45S5 x = 60 wt.%	T = 1000 °C, t = 5 h	Composites of β-TCP/45S5 after sintering included NaCaPO_4_, CaSiO_3_, and amorphous phase. The composite’s surface revealed a spongious bone-like morphology after treatment with each acid (aggressive level: HCl > H_2_SO_4_ > H_2_SO_4_-CrO_3_ ≈ HNO_3_).	Composites containing non-covalently immobilized rhBMP-2 on the surface exhibited significant biological activity in contrast to the composites with covalently bound protein on the surface.	[108]
**SLS**	(100 − x)TCP/x45S5 x = 1, 2.5, 5, 10, 15 wt.%	T = 1100 °C, heating rate = 0.5 °C/min, cooled rate = 0.5 °C/min, t = 3 h	Composites of β-TCP/45S5 with x ≤ 5 wt.% after sintering included β-TCP and α-TCP; with x > 5 wt.% included β-CaSiO_3_ and NaCaPO_4_. The mechanical properties (fracture toughness, compressive strength, and stiffness values) increased with an increase in 45S5 from 0 to 5 wt.%, reached maximum values (1.67 MPam^1/2^, 21.32 MPa, and 264.32 MPa) at 5 wt.%, and then decreased with further increase in 45S5 to 15 wt.%.	SBF (14 days of immersion) test showed homogeneous apatite layer formation for composites with x ≥ 5 wt.%. The MG-63 cells were able to attach, spread, and proliferate safely without toxic interference from the composites, especially with x = 5 wt.%.	[109]
**DLP**	(100 − x)BCP/x45S5 x = 20 wt.% BCP = HAp:TCP = 6:4	T = 1200 °C, t = 2, 4, 6 h	Composites after sintering for 2 h included α-TCP, CaSiO_3_, and Na_2_CaSiO_4_; with exposure for 4 and 6 h included α-TCP, CaSiO_3_, Ca_5_(PO_4_)_2_SiO_4_, and Na_2_Ca_3_Si_6_O_16_. The greater the holding time was, the more growth and roughening of ceramic grains were observed. The best compressive strength was 1.735 MPa at the holding time of 4 h.	SBF (60 h immersion) test showed composite bioceramic with 20 wt.% 45S5 had better bioactivity than pure BCP.	[110]

***Abbreviations***: 45S5—Bioglass 45S5; ALP—alkaline phosphatase; BCP—biphasic calcium phosphate; *C. albicans*—*Candida albicans;* COL-1A—collagen type 1 alpha 1 chain; DLP—digital light processing; *E. coli*—*Escherichia coli*; hADMSCs—human-adipose-derived mesenchymal stem cells; HAp—hydroxyapatite; hBMSCs—human bone marrow mesenchymal stem cells; hFOB—human fetal osteoblasts; MG-63—human osteosarcoma cells line; rhBMP-2—recombinant human bone morphogenetic proteins 2; RUNX-2—runt-related transcription factor 2; *S. aureus*—*Staphylococcus aureus*; SBF—simulated body fluid; SLS—selective laser sintering; SPP-1—secreted phosphoprotein 1; TCP—tricalcium phosphate.

## Data Availability

Not applicable.
